# Antipsychotic prescribing patterns during and after critical illness: a prospective cohort study

**DOI:** 10.1186/s13054-016-1557-1

**Published:** 2016-11-24

**Authors:** Jason E. Tomichek, Joanna L. Stollings, Pratik P. Pandharipande, Rameela Chandrasekhar, E. Wesley Ely, Timothy D. Girard

**Affiliations:** 1 Department of Pharmaceutical Services, Vanderbilt University Medical Center, 1211 Medical Center Drive, Nashville, TN 37232-7610 USA; 2 Department of Anesthesiology, Division of Critical Care, Vanderbilt University School of Medicine, 1211 21st Ave S, Nashville, TN 37212 USA; 3 Department of Biostatistics, Vanderbilt University School of Medicine, 2525 West End Avenue, Nashville, TN 37203 USA; 4 Division of Allergy, Pulmonary, and Critical Care Medicine, Department of Medicine, Vanderbilt University School of Medicine, 1161 21st Ave S, Nashville, TN 37232-2650 USA; 5 Center for Health Services Research, Vanderbilt University School of Medicine, 1215 21st Ave S, Nashville, TN 37232-8300 USA; 6 Anesthesia Service, Department of Veterans Affairs Medical Center, Tennessee Valley Healthcare System, 1310 24th Ave S, Nashville, TN 37212 USA; 7 Geriatric Research, Education and Clinical Center (GRECC) Service, Department of Veterans Affairs Medical Center, Tennessee Valley Healthcare System, 1310 24th Ave S, Nashville, TN 37212 USA; 8 Clinical Research, Investigation, and Systems Modeling of Acute illness (CRISMA) Center, Department of Critical Care Medicine, University of Pittsburgh School of Medicine, 3550 Terrace Street, Pittsburgh, PA 15261 USA

**Keywords:** Antipsychotic agents, Critical care, Delirium

## Abstract

**Background:**

Antipsychotics are used to treat delirium in the intensive care unit (ICU) despite unproven efficacy. We hypothesized that atypical antipsychotic treatment in the ICU is a risk factor for antipsychotic prescription at discharge, a practice that might increase risk since long-term use is associated with increased mortality.

**Methods:**

After excluding patients on antipsychotics prior to admission, we examined antipsychotic use in a prospective cohort of ICU patients with acute respiratory failure and/or shock. We collected data on medication use from medical records and assessed patients for delirium using the Confusion Assessment Method for the ICU. Using multivariable logistic regression, we analyzed whether age, delirium duration, atypical antipsychotic use, and discharge disposition (each selected a priori) were independent risk factors for discharge on an antipsychotic. We also examined admission Acute Physiology and Chronic Health Evaluation (APACHE) II score, haloperidol use, and days of benzodiazepine use in post hoc analyses.

**Results:**

After excluding 18 patients due to prior antipsychotic use and three who withdrew, we included 500 patients. Among 208 (42%) treated with an antipsychotic, median (interquartile range) age was 59 (49–69) years and APACHE II score was 26 (22–32), characteristics that were similar among antipsychotic nonusers. Antipsychotic users were more likely than nonusers to have had delirium (93% vs. 61%, *p* < 0.001). Of the 208 antipsychotic users, 172 survived to hospital discharge, and 42 (24%) of these were prescribed an antipsychotic at discharge. Treatment with an atypical antipsychotic was the only independent risk factor for antipsychotic prescription at discharge (odds ratio 17.6, 95% confidence interval 4.9 to 63.3; *p* < 0.001). Neither age, delirium duration, nor discharge disposition were risk factors (*p* = 0.11, 0.38, and 0.12, respectively) in the primary regression model, and post hoc analyses found APACHE II (*p* = 0.07), haloperidol use (*p* = 0.16), and days of benzodiazepine use (*p* = 0.31) were also not risk factors for discharge on an antipsychotic.

**Conclusions:**

In this study, antipsychotics were used to treat nearly half of all antipsychotic-naïve ICU patients and were prescribed at discharge to 24% of antipsychotic-treated patients. Treatment with an atypical antipsychotic greatly increased the odds of discharge with an antipsychotic prescription, a practice that should be examined carefully during medication reconciliation since these drugs carry “black box warnings” regarding long-term use.

## Background

Despite a dearth of evidence supporting their use, antipsychotics have long been preferred agents for the treatment of delirium in the intensive care unit (ICU). Clinical practice guidelines published in 2002 by the Society of Critical Care Medicine (SCCM) recommended haloperidol based on clinical experience and theoretical benefit [1]; antipsychotics can improve the positive symptoms of psychosis, many of which are similar to symptoms of hyperactive delirium. Numerous surveys have documented ICU practitioners’ preference for haloperidol or atypical antipsychotics when treating delirium [2–4]. Yet, in the updated SCCM guidelines on the management of pain, agitation, and delirium published in 2013 [5], the authors concluded that there was no evidence supporting the use of haloperidol to treat delirium in the ICU, and the use of atypical antipsychotics was supported only by low-quality evidence.

The use of antipsychotics, especially for prolonged periods of time, is not without risk. Side effects include ventricular arrhythmias, excess sedation, akathisia, and hypotension, among others [6]. Indeed, long-term use of antipsychotics among vulnerable populations may increase the risk of death [7, 8], and these drugs now carry “black box warnings” from the US Food and Drug Administration (FDA) regarding treatment of dementia-related psychosis in elderly patients. The risk-to-benefit ratio for antipsychotics initiated in the ICU may therefore increase sharply if they are continued after hospital discharge, a practice noted in recent studies to affect up to one-third of treated patients [9–12]. During medication reconciliation, which plays a key role in the hospital discharge process, clinicians will ideally discontinue antipsychotics that were started in the ICU for delirium (especially if delirium has resolved), but they may be reluctant to do so if intensivists continued the antipsychotic upon transfer out of the ICU. Knowledge of risk factors for being discharged on an antipsychotic initiated for delirium in the ICU may contribute to more informed decision making.

We conducted an observational study to identify ICU patients most likely to be discharged from the hospital with a prescription for an antipsychotic (typical or atypical). Based on our clinical observation that patients discharged on an antipsychotic are nearly always prescribed an atypical rather than typical antipsychotic, we hypothesized that atypical antipsychotic treatment started in the ICU is an independent risk factor for discharge on an antipsychotic. We also hypothesized that older age, prolonged delirium, and discharge to a location other than home would be independently associated with discharge on an antipsychotic.

## Methods

### Study design and population

We embedded this single-center, prospective cohort study in the Bringing to Light the Risk Factors and Incidence of Neuropsychological Dysfunction in ICU Survivors (BRAIN-ICU) Study [13], which enrolled patients in two hospitals in Nashville, TN, USA, from March 2007 to May 2010. All BRAIN-ICU subjects enrolled at Vanderbilt University Medical Center were eligible for the current investigation of discharge on antipsychotics except those who were receiving antipsychotics prior to hospital admission. Adults treated in a medical or surgical ICU for respiratory failure and/or shock were enrolled in the BRAIN-ICU Study unless they met one or more of the following exclusion criteria, which have been described in detail elsewhere [13]: recent substantial critical illness requiring ICU admission, conditions that would make assessments for delirium unreliable (e.g., deafness, blindness, etc.), conditions that would prohibit long-term follow-up, life expectancy <24 h, lack of informed consent, and known or suspected severe neurologic disease. The study protocol was approved by the Vanderbilt University Institutional Review Board.

### Clinical characteristics

We collected data on demographics and clinical characteristics from the electronic medical record at the time of enrollment and throughout the patient’s hospital stay. These included patient age, gender, ethnicity, chronic disease burden as per the Charlson Comorbidity Index [14], severity of illness as per the Acute Physiology and Chronic Health Evaluation (APACHE) II score [15] and Sequential Organ Failure Assessment (SOFA) [16] score, ICU type, admission diagnosis, duration of mechanical ventilation, ICU and hospital length of stay, and receipt of antipsychotics as well as sedatives and analgesics. Trained research personnel assessed patients for delirium twice daily while they were in the ICU and once daily after ICU discharge using the Confusion Assessment Method for the ICU (CAM-ICU) [17, 18]. We considered any day during which at least one CAM-ICU assessment was positive to be a day of delirium and summed these days to determine delirium duration. Discharge disposition (e.g., home, long-term acute care hospital, nursing home, etc.) was extracted from the discharge orders recorded in the medical record.

### Outcome

We used discharge summaries and pharmacy records to identify antipsychotics prescribed at the time of hospital discharge. Specifically, we considered an antipsychotic to be prescribed at discharge if it was included in the patient’s discharge medication summary and/or a new outpatient prescription for the antipsychotic was recorded in the electronic pharmacy records.

### Statistical analysis

We examined baseline demographics and clinical characteristics using median and interquartile range (IQR) for continuous variables and proportions for categorical variables. To compare patients who were treated with an antipsychotic with those who were not in bivariate analyses, we used the chi-squared test for categorical variables and the Wilcoxon-Mann-Whitney test for continuous variables. We also used these tests in bivariate analyses comparing patients discharged on an antipsychotic with those who received an antipsychotic in the hospital but were not discharged on the medication, i.e., the antipsychotic was discontinued prior to discharge.

We used multivariable logistic regression to determine whether a priori-selected risk factors were independently associated with discharge on an antipsychotic. To avoid model overfitting, we limited the number of covariates examined in our primary regression model by adhering to the rule of thumb that a multivariable model must fit no more than *m*/10 parameters (where *m* is the effective sample size) for it to be reliable in similar cohorts [19]. Since 42 patients were discharged on an antipsychotic (*m* = 42), we included four (42/10) covariates in our primary regression model: age, delirium duration, atypical antipsychotic use, and discharge disposition. Additionally, in light of results of a recent study reporting that admission APACHE II score and duration of benzodiazepine treatment were independent risk factors for discharge on an antipsychotic [10], we also conducted a post hoc analysis by including these covariates (one at a time) in logistic regression models with the four covariates previously specified. Finally, we also added haloperidol use to the regression model in a post hoc analysis prompted by the very strong bivariate association observed between haloperidol use and discharge on an antipsychotic. We used Stata version 10.1 for all statistical analyses and considered a two-sided alpha of 0.05 to indicate statistical significance.

## Results

**Fig. 1:**
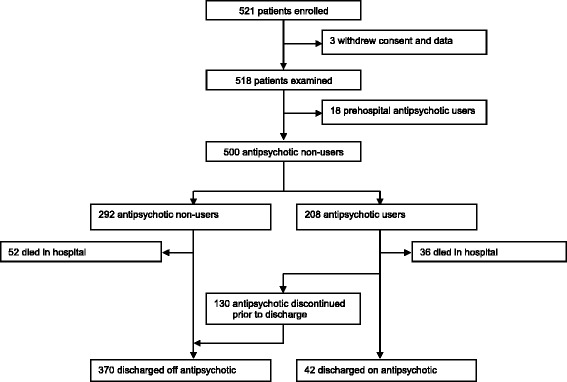
Patient Flowchart. Treatment with antipsychotics during hospitalization and at the time of hospital discharge is shown

Of 521 patients enrolled in the BRAIN-ICU study at Vanderbilt, we excluded 18 from the current study because they were antipsychotic users prior to hospital admission and three who withdrew after enrollment; 500 patients were therefore included in this study of antipsychotic prescribing (Fig. 1). Of these, 208 (42%) were newly treated with an antipsychotic in the ICU. This included adults of all ages, with the majority having a high severity of illness, and most being admitted to the ICU with sepsis/acute respiratory distress syndrome (ARDS) (37%), after surgery (21%), or with a cardiac illness (11%), characteristics that were similar among those who did not receive an antipsychotic (Table 1). New antipsychotic users were significantly more likely than nonusers to have been mechanically ventilated at enrollment (93% vs. 83%, *p* = 0.002), to have had delirium (93% vs. 61%, *p* < 0.001), and to have experienced a longer duration of delirium (1 vs. 5 days, *p* < 0.0001).

**Table 1:** Demographics and clinical characteristics

Characteristics^a^	Antipsychotic non-users *N* = 292	Antipsychotic users *N* = 208	*p*
Age, years	58 (45–66)	59 (49–69)	0.14
Female, *N* (%)	141 (48.3)	95 (45.7)	0.56
Caucasian, *N* (%)	248 (84.9)	191 (91.8)	0.08
Baseline cognitive impairment, *N* (%)^b^	30 (10.3)	29 (13.9)	0.21
Charlson Comorbidity Index	2 (1–4)	2 (1–4)	0.33
APACHE II score at study enrollment	25 (18–31)	26 (22–32)	0.10
SOFA score at study enrollment	9 (7–13)	10 (7–12)	0.52
ICU type, *N* (%)			0.22
Medical	183 (62.7)	119 (57.2)	
Surgical	109 (37.3)	89 (42.8)	
ICU admission diagnoses, *N* (%)			0.29
Sepsis/ARDS	83 (28.4)	77 (37.0)	
Surgery^c^	65 (22.2)	43 (20.7)	
CHF/MI/arrhythmia	59 (20.2)	23 (11.1)	
Altered mental status	37 (12.7)	26 (12.5)	
COPD/asthma	14 (4.8)	5 (2.4)	
Other^d^	34 (11.6)	34 (16.3)	
Mechanical ventilation at enrollment, *N* (%)	243 (83.2)	193 (92.8)	0.002
ICU length of stay, days	3 (2–6)	9 (4–14)	<0.0001
Delirium			
Prevalence during study period, *N* (%)	178 (61.0)	193 (92.8)	<0.0001
Duration, days	1 (0–3)	5 (2–8)	<0.0001

^a^Values are shown as median (interquartile range) unless otherwise noted

^b^Patients with a Short IQCODE score ≥3.3 were considered to have pre-existing cognitive impairment of mild to moderate severity. We excluded patients from study enrollment with dementia that prevented them from living independently and/or with a Clinical Dementia Rating (CDR) score of 3 or more, indicative of severe dementia

^c^Excluding cardiac and neurological surgery

^d^Including cirrhosis/hepatic failure, gastrointestinal bleeding, malignancy, metabolic disarray, hemoptysis, pulmonary embolism, renal failure, and seizure/status epilepticus

*ARDS* acute respiratory distress syndrome, *ADL* activities of daily living, *APACHE II* Acute Physiology and Chronic Health Evaluation II, *CHF* congestive heart failure, *COPD* chronic obstructive pulmonary disease, *IQCODE* Informant Questionnaire of Cognitive Decline in the Elderly, *IADL* instrumental activities of daily living, *MI* myocardial infarction, *ICU* intensive care unit, *SOFA* Sequential Organ Failure Assessment

Of the 172 antipsychotic users who survived to hospital discharge, 42 (24%) were prescribed an antipsychotic at discharge. Only seven of these patients were delirious at the time of hospital discharge; 28 had a normal mental status, one was comatose, and six had an unknown mental status because they were discharged after the 30-day period of study assessments and medical records did not clearly document whether or not delirium was present at discharge.

In unadjusted comparisons, patients whose antipsychotics were discontinued prior to discharge generally had characteristics similar to those among patients who were prescribed antipsychotics at discharge, with the exception that patients prescribed antipsychotics at discharge were much more likely to have received an atypical antipsychotic and less likely to have received haloperidol during their hospital stay (both *p* < 0.001; Table 2). Among the 100 patients who survived to discharge after treatment with an atypical antipsychotic, 82 (82%) received olanzapine during their hospital stay, 5 (5%) received risperidone, 12 (12%) received quetiapine, and 15 (15%) received ziprasidone; 14 (14%) of these patients received more than one atypical antipsychotic during their hospital stay.

**Table 2:** Characteristics according to discharge on antipsychotic

Characteristics^a^	Antipsychotic discontinued before discharge *N* = 130	Antipsychotic prescribed at discharge *N* = 42	*p*
Age, years	57 (48–69)	60 (52–71)	0.20
Female, *N* (%)	61 (46.9)	21 (50)	0.73
Caucasian, *N* (%)	122 (93.9)	38 (90.5)	0.49
Baseline cognitive impairment, *N* (%)^b^	20 (15.4)	7 (16.7)	0.84
Charlson Comorbidity Index	2 (1–4)	2 (1–3)	0.35
ICU type, *N* (%)			0.55
Medical	75 (57.7)	22 (52.4)	
Surgical	55 (42.3)	20 (47.6)	
ICU length of stay, days	7 (4–14)	9 (4–12)	0.94
Delirium			
Prevalence during study period, *N* (%)	120 (92)	40 (95)	0.52
Duration, days	4 (2–8)	6 (3–9)	0.12
Antipsychotic received, *N* (%)^c^			
Atypical	61 (46.9)	39 (92.9)	<0.001
Haloperidol	106 (81.5)	23 (54.8)	<0.001
Discharge disposition, *N* (%)			0.08
Home	60 (46.2)	12 (28.6)	
Long-term acute care hospital	13 (10.0)	6 (14.3)	
Rehabilitation facility	38 (29.2)	15 (35.7)	
Nursing home	7 (5.4)	6 (14.3)	
Other	12 (9.2)	3 (7.1)	

^a^Values are shown as median (interquartile range) unless otherwise noted

^b^Patients with a Short IQCODE score ≥3.3 were considered to have pre-existing cognitive impairment of mild to moderate severity. We excluded patients from study enrollment with dementia that prevented them from living independently and/or with a Clinical Dementia Rating (CDR) score of 3 or more, indicative of severe dementia

^c^Percentages sum to >100% because 50 patients received two different antipsychotics, and 14 patients received three different antipsychotics

*ICU* intensive care unit, *IQCODE* Informant Questionnaire of Cognitive Decline in the Elderly

Among patients treated with an antipsychotic in the hospital, receipt of an atypical antipsychotic was the only independent risk factor for discharge on an antipsychotic in our primary regression model (odds ratio (OR) 17.6, 95% confidence interval (CI) 4.9 to 63.3, *p* < 0.001; Table 3). When added to the regression model in post hoc analyses, neither admission APACHE II score (OR 0.95, 95% CI 0.90 to 1.00, *p* = 0.07), days of benzodiazepine use (OR 0.95, 95% CI 0.87 to 1.04, *p* = 0.31), nor haloperidol use (OR 0.51, 95% CI 0.20 to 1.30, *p* = 0.16) were associated with discharge on an antipsychotic, and atypical antipsychotic use remained a strong independent risk factor (*p* < 0.001 in all models) after adjusting for these additional covariates.

**Table 3:** Risk factors for discharge on antipsychotic medication

Risk factor	Odds ratio	95% Confidence interval	*p*
Age, years	1.02	0.99 to 1.05	0.11
Atypical antipsychotic received, yes/no	17.6	4.9 to 63.3	<0.001
Delirium duration, days	1.03	0.97 to 1.10	0.38
Discharge to home, yes/no	0.98	0.95 to 1.01	0.12

## Discussion

In this prospective cohort study, we found that nearly half of all antipsychotic-naïve patients admitted with critical illness were treated with one or more antipsychotics during their ICU stay, and one out of every four antipsychotic-treated patients was discharged on an antipsychotic even though the majority were no longer delirious and these drugs carry “black box warnings” from the FDA. In contrast to receipt of haloperidol, which was not associated with discharge on an antipsychotic, treatment with an atypical antipsychotic was independently associated with an increase in the odds of discharge on an antipsychotic, suggesting that the use of atypical antipsychotics in the ICU may place an important subset of patients at increased risk for adverse effects after resolution of critical illness. Future studies are needed to determine not only the risk-benefit ratio of antipsychotic use during critical illness but also the long-term effects of these medications in this increasingly exposed patient population.

Delirium—characterized by acute changes and fluctuations in mental status accompanied by inattention, disorganized thinking, and/or an altered level of consciousness [20]—is common in the ICU, where up to 60% to 80% of mechanically ventilated patients are affected. Given that patients with delirium are at increased risk for agitation-related adverse outcomes as well as mortality [21–23], longer hospital stays [24], and long-term cognitive impairment [13, 25], clinicians frequently treat delirium with antipsychotics, a practice that was recommended in older clinical practice guidelines [1]. These medications may offer some short-term benefit by reducing agitation [26], but their effect on delirium and associated outcomes (including long-term cognitive function) remains unclear.

To date, only three randomized, placebo-controlled trials—ranging in size from 36 to 142 subjects—have examined the efficacy of antipsychotics for the management of delirium in the ICU. In a trial of 101 mechanically ventilated ICU patients [27], we examined haloperidol vs. ziprasidone vs. placebo for the prevention and treatment of delirium and found that neither antipsychotic changed the number of days that patients were alive without delirium or coma (*p* = 0.66). Similarly, Page and colleagues [26] compared haloperidol with placebo in 142 mechanically ventilated ICU patients and found that haloperidol had no effect on days alive without delirium or coma (*p* = 0.53). In contrast, Devlin et al. [28] compared quetiapine with placebo for the treatment of persistent delirium in 36 ICU patients who had not responded to haloperidol and found that quetiapine reduced the duration of delirium (*p* = 0.006) and of agitation (*p* = 0.02). Given the small sample sizes and conflicting results of these trials, adequately powered, multicenter, randomized, placebo-controlled trials are needed to determine whether antipsychotics are beneficial before this class of medications can be broadly recommended for the treatment of delirium in the ICU.

Medications started during a hospital stay are often continued at the time of hospital discharge, a care transition when patients are at high risk of medication errors. Indeed, both unintentional medication discrepancies—which affect up to 70% of patients at hospital discharge [29]—and the intentional prescription of potentially inappropriate medications [30] place recovering patients at risk for serious adverse drug events at a time when they are still highly vulnerable yet less closely monitored. Antipsychotics are not the only class of medications that are prescribed at discharge despite being no longer indicated at that time. Scales and colleagues [31] examined four medication classes, for example, and found that all four were prescribed at hospital discharge without a documented indication. We specifically focused on antipsychotics in the current study because, unlike some medications (e.g., gastric acid suppressants), antipsychotics have never been proven efficacious in the ICU setting and their adverse effects during chronic use are well documented. From 1995 to 2008, the off-label use of antipsychotic medications nearly doubled despite warnings from the FDA and others regarding serious risk [32]. It is unknown how much of this increase in outpatient atypical antipsychotic use is related to the increase in prescribing of atypical antipsychotics for delirium in the ICU.

Our results advance and extend a growing body of evidence showing that antipsychotics initially prescribed for delirium in the ICU are often continued at the time of hospital discharge. In a retrospective cohort study, Jasiak et al. [9] found that 20 (34%) of 59 patients treated for delirium with an antipsychotic in the ICU were discharged from the hospital on the medication, a practice they estimated would increase outpatient medication costs by more than $2225 per patient per year. Several other studies have subsequently reported that 21–29% [10–12] of patients started on antipsychotics in the ICU were discharged with a prescription to continue the antipsychotic, findings almost identical to ours. Taken together with results from a multicenter study of 164,996 hospitalizations across 71 academic medical centers in the US [33], which reported that 1 in 10 ICU patients received an antipsychotic during their hospital stay, these data suggest that approximately 2.5% of the >5 million patients discharged from US ICUs each year are prescribed an antipsychotic at discharge that they were not taking prior to their critical illness.

Our findings compliment those of other recent studies that examined independent risk factors for discharge on an antipsychotic started in the ICU. Rowe et al. [10] found higher severity of illness (measured via APACHE II score at ICU admission) and longer duration of benzodiazepine sedation were associated with discharge on an antipsychotic. Perhaps due to different practice patterns, we found no association between these factors and antipsychotic prescription at discharge in post hoc analyses. Though atypical antipsychotic use was not among the variables analyzed by Rowe et al., Marshall and colleagues [12] reported results consistent with ours—ICU patients treated with quetiapine or olanzapine were significantly more likely to be prescribed an antipsychotic at discharge than patients who did not receive an atypical antipsychotic in the ICU. One might assume that treatment with an atypical antipsychotic is a prerequisite for discharge on an antipsychotic, but this is not the case since haloperidol—the most commonly prescribed antipsychotic in the ICU—is available as an oral preparation. Yet, only atypical antipsychotic use (and not haloperidol use) was independently associated with discharge on an antipsychotic in our study, a finding suggesting that a potential disadvantage of using an atypical antipsychotic when treating delirium in the ICU is increased risk of inappropriate use after discharge. In a study examining medications prescribed at hospital discharge to 120 elderly critical illness survivors, we found that 71% of atypical antipsychotics prescribed were deemed inappropriate by an expert panel comprised of a geriatrician, a hospitalist, and a clinical pharmacist [30].

Strengths of this study include a prospective study design, carefully characterized risk factors including delirium assessed using the well-validated CAM-ICU, and use of multivariable regression to identify independent risk factors for discharge on an antipsychotic. The study also has important limitations. As a single-center study, its findings may be generalizable only to hospitals with patients and providers similar to those in our medical center. At the time of this investigation, for example, Vanderbilt Medical Center was not yet using the electronic discharge wizard now employed to facilitate medication reconciliation. Thus, our results may be more applicable in hospitals without formal medication reconciliation practices. Given the observational study design and the modest sample size, which limited the number of covariates we could reliably fit in the regression model, we cannot rule out confounding by variables not included nor can we rule out type II error leading to a negative finding regarding discharge location, which was associated with antipsychotic prescription at discharge in one recent study of ICU patients [12]. Additionally, without clear documentation by providers of the reasons they prescribed antipsychotics at hospital discharge, we cannot ascertain the rationale for treatment decisions at discharge. Some clinicians, for example, may have chosen to prescribe antipsychotics at discharge due to an anticipated benefit, whereas lack of familiarity could also make clinicians reluctant to discontinue a medication started by another provider. Our findings may overestimate the frequency of antipsychotic use after discharge since we measured prescriptions for antipsychotics but did not confirm post-discharge adherence to these prescriptions. Finally, the extent of long-term use and any resultant adverse effects could not be determined because of the lack of follow-up after hospital discharge.

## Conclusion

In a large cohort of patients recovering from critical illness, antipsychotics were prescribed at hospital discharge to one out of every four patients newly treated with antipsychotics for delirium in the ICU, a practice most likely to occur among patients treated with an atypical antipsychotic in the hospital. Not only are the efficacy and safety of antipsychotics for delirium in the ICU unproven, but it remains unclear which antipsychotic, if any, should be used to treat delirium and for how long. Until clear evidence from large randomized trials is available regarding the efficacy and appropriate duration of antipsychotic use for delirium in the ICU, this class of medication should be used with caution. Additionally, focused efforts should be implemented to ensure antipsychotics are appropriately discontinued upon transitions of care in the hospital.

## Key messages

- Nearly half of all antipsychotic-naïve patients admitted with critical illness were treated with an antipsychotic during their ICU stay.
- One out of every four patients newly treated with an antipsychotic for delirium in the ICU was discharged on an antipsychotic.
- Treatment with an atypical antipsychotic in the hospital was independently associated with a 17-fold increase in the odds of discharge on an antipsychotic.
- Treatment with haloperidol was not associated with discharge on an antipsychotic after adjusting for atypical antipsychotic use.

## Abbreviations

APACHE: Acute Physiology and Chronic Health Evaluation
CAM: Confusion Assessment Method
FDA: Food and Drug Administration
ICU: Intensive care unit
IQR: Interquartile range
SCCM: Society of Critical Care Medicine
SOFA: Sequential Organ Failure Assessment
